# Cell fate changes induced by a *Distal-less* enhancer-trap transgene in the *Drosophila* antennal imaginal disc

**DOI:** 10.1038/s41598-018-23093-z

**Published:** 2018-03-21

**Authors:** Syeda Nayab Fatima Abidi, Rachel K. Smith-Bolton

**Affiliations:** Department of Cell and Developmental Biology, University of Illinois Urbana-Champaign, Urbana, IL 61801 USA

## Abstract

The imaginal discs of the genetically tractable model organism *Drosophila melanogaster* have been used to study cell-fate specification and plasticity, including homeotic changes and regeneration-induced transdetermination. The identity of the reprogramming mechanisms that induce plasticity has been of great interest in the field. Here we identify a change from antennal fate to eye fate induced by a *Distal-less-GAL4 (DllGAL4)* P-element insertion that is a mutant allele of *Dll* and expresses GAL4 in the antennal imaginal disc. While this fate change is not induced by tissue damage, it appears to be a hybrid of transdetermination and homeosis as the GAL4 expression causes upregulation of Wingless, and the *Dll* mutation is required for the fate change. Neither GAL4 expression nor a *Dll* mutation on its own is able to induce antenna-to-eye fate changes. This plasticity appears to be unique to the *DllGAL4* line, possibly due to cellular stress induced by the high GAL4 expression combined with the severity of the *Dll* mutation. Thus, we propose that even in the absence of tissue damage, other forms of cellular stress caused by high GAL4 expression can induce determined cell fates to change, and selector gene mutations can sensitize the tissue to these transformations.

## Introduction

Normal development requires that cells become progressively restricted in their potential as they become determined and differentiate toward specific fates. This determination of fate is regulated by homeotic or selector genes^1^. *Drosophila* imaginal discs, precursors of adult fly appendages, are an important system for studying fate determination and developmental plasticity. In *Drosophila*, the imaginal disc primordia are established and acquire a disc-specific determined state during embryogenesis^2–4^. The discs grow and maintain their determined state through the larval stages and then proceed through differentiation during metamorphosis. This state of determination with respect to disc type usually stays fixed. However, fate determination is not completely irreversible and two processes can produce changes in disc identity: homeosis and transdetermination.

Homeosis occurs when mutations are generated in hox or selector genes, which are important for establishing cell, tissue and segment identities. These mutations result in replacement of one body part by another^5^. Transdetermination occurs when cells or tissues switch from one determined state to another due to experimental manipulation. Mechanically fragmented imaginal discs cultured in adult hosts have the ability to regenerate and can faithfully replace the lost structures over many generations of serial fragmentation and culture, indicating that the determined state is maintained^6,7^. However, in specific locations in each disc, called the weak point, a few cells become plastic and undergo transdetermination^8^. While all imaginal discs are capable of transdetermination, this fate change is not a random event, occurring only in particular reproducible directions with characteristic probabilities. The restricted nature of these transformations indicates that while disc determination is plastic, disc cells prefer certain developmental pathways over others.

In addition to tissue damage, ectopic activation of different signaling pathways, such as Wingless (Wg), Decapentaplegic (Dpp), and Jun N-terminal Kinase (JNK) signaling, can also induce transdetermination events. Misexpression of *wingless* in the foreleg imaginal discs induces transdetermination to wing cells in a manner very similar to fragmentation^9^. Dpp signaling has an important role in defining the weak point, and high levels of endogenous *dpp* expression enable transdetermination in response to both damage-induced and transgene-induced ectopic *wingless* expression^10^. Maves and Schubiger have proposed that the ectopic interaction of Wg and Dpp signaling in wounded imaginal discs induces transdetermination at the points where they overlap^11^. Furthermore, JNK signaling, which is activated upon wounding^12,13^ can induce transdetermination through the suppression of Polycomb group (PcG) proteins^14^.

There has been much debate in the literature over whether homeotic transformations and transdetermination are different aspects of the same phenomenon or are distinct processes^15,16^. Despite the different causes of the two phenomena, tissue damage and mutations, they share similarities. Transdetermination was predicted to alter expression of genes that act as developmental switches^17^, and subsequent work showed that homeotic gene expression is altered in transdetermining tissue^18^. Additionally, misexpression of selector genes can result in transformations that phenotypically resemble transdetermination^5,19^. It remains to be seen whether cell fate plasticity can be induced through mechanisms other than tissue damage, such as general cellular stress, mechanical tension, or activation of alternative signaling pathways.

To support the possibility of damage-independent plasticity, here we describe an antenna-to-eye fate change that occurs in response to GAL4 expression. We found a *Distal-less (Dll)* GAL4 line, which is also a mutant for the *Dll* gene, that causes apoptosis in the *Distal-less* domain in a temperature-dependent manner. This apoptosis induced many aspects of the damage response, including expansion of the Wg expression domain, as well as upregulation of JNK signaling and compensatory proliferation. The GAL4 expression also led to certain cells in the third antennal segment changing fate to produce pigmented eye tissue. Surprisingly, caspase-mediated cell death was not required for the fate change, suggesting it was not induced by tissue damage. The fate change was also not due to just the *Dll* mutation or just GAL4 expression, because neither *Dll* mutations nor GAL4 expression alone were able to produce this particular fate change. Furthermore, other *Dll* mutations in combination with a different GAL4 transgene that was expressed in the same cells (*ssGAL4)* caused some tissue disruption, but did not induce the fate change. Thus, the fate change appears to be specific to the *DllGAL4* line, where the unique combination of the strength of *DllGAL4* expression and severity of the *Dll* mutation may cause enough cellular stress to perturb cell fates. Therefore, cellular stress, independent of cell death, could play a role in perturbing cell fate in a sensitized mutant background. This stress-induced plasticity seems to be a hybrid of transdetermination and homeosis, and may confound interpretation of experiments conducted using this *DllGAL4* line for analysis of different developmental processes.

## Results

### High expression of GAL4 induces cell death at elevated temperatures

**Figure 1:**
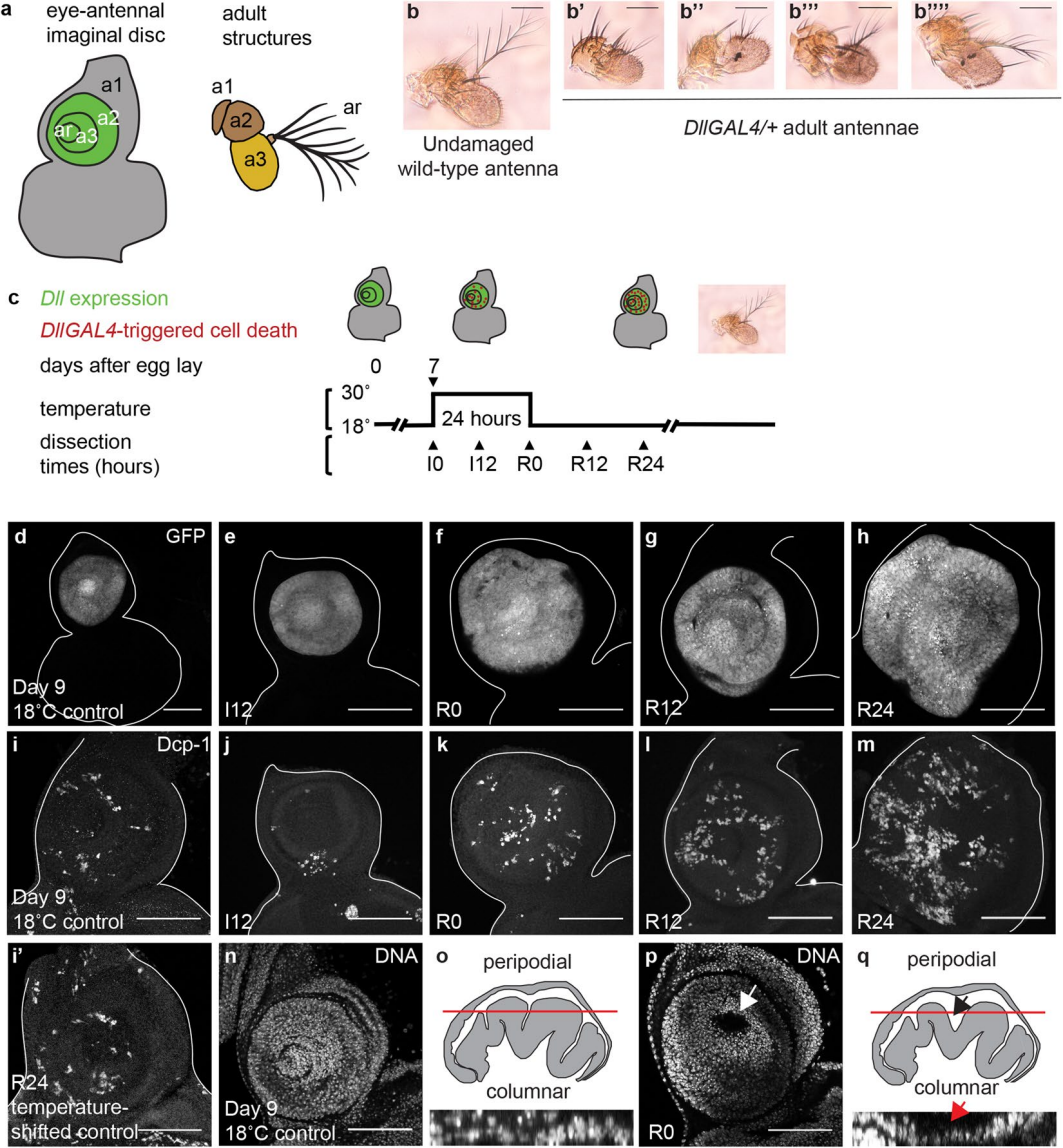
High *DllGAL4* expression induces cell death in the antennal disc. (**a**) Diagram showing regions of the eye-antennal imaginal disc and corresponding adult structures. a1, a2, a3 and ar are first, second, and third antennal segments and the arista, respectively. The green region shows *Distal-less* expression. (**b**) Undamaged wild-type adult antenna. (**b’**–**b””**) Adult antennae from *DllGAL4*/+ animals raised at 25 °C. The antennae showed a range of altered morphology. The aristae and the third antennal segments were the most affected. (**c**) The protocol used to observe *DllGAL4*-induced phenotypes. Animals were raised at 18 °C and shifted to 30 °C for 24 hours during early third-instar larval development (day 7 AEL). Larvae were returned to 18 °C and allowed to pupariate and eclose or were dissected at the time points noted during damage induction (I) or recovery (R). (**d**–**h**) *DllGAL4* expression marked by *UAS-EGFP* in a day 9 18 °C control disc (*DllGAL4*/+), which is developmentally similar to R0 (**d**), and temperature-shifted *DllGAL4*/+ discs at I12 (**e**), R0 (**f**), R12 (**g**), and R24 (**h**). (**i** and **i’**) Dying cells marked using an antibody for the cleaved form of the effector caspase Dcp-1 in a day 9 18 °C control disc (*DllGAL4*/+) (**i**) and a temperature-shifted control disc (*w*^1118^; +/*SM6.TM6B*) at R24 (**i’**). Note that there was no temperature-induced increase in cleaved Dcp-1 positive cells in these control discs. (**j**–**m**) A progressive increase in dying cells was seen during induction and recovery times as marked by cleaved Dcp-1 immunostaining. Note that I12 discs had less apoptosis compared to control discs, due to I12 discs being developmentally younger. (**n** and **p**) Disc morphology was affected, as visualized with nuclear staining of the antennal epithelium using TO-PRO-3 in day 9 18 °C control discs (**n**) and R0 discs (**p**). In (**p**) the arrow marks the gap between the peripodial and columnar epithelia. (**o**) Orthogonal view of part of the disc shown in (**n**) and diagram of a cross-section of an undamaged antennal disc. The red line shows the approximate level of the confocal image of the undamaged epithelium shown in (**n**). (**q**) Orthogonal view of part of the disc shown in (**p**) and diagram of a cross-section of an R0 disc. The arrows indicate the gap between the epithelial layers visible in (**p**). Scale bars are all 100 μm.

To express transgenes in the antennal imaginal disc for experimental purposes, we used a GAL4 enhancer trap in the *Distal-less (Dll)* locus^20^, which is expressed in the arista and the second and third antennal segments (Fig. 1a). In the course of these experiments, we examined the *DllGAL4* line independently as a control. Remarkably, the *DllGAL4* alone produced antennal phenotypes. *DllGAL4* animals that were maintained at 18 °C rarely showed antennal defects, while animals maintained at 25 °C or shifted to 30 °C during early third instar for 24 hours showed a high frequency of defects, including altered morphology in the arista and the third antennal segment (Fig. 1b–b””). Importantly, GAL4 activity is increased at higher temperatures, which could explain the morphological defects at 25 °C and 30 °C^21^. *DllGAL4* animals kept at 18 °C throughout development were used as controls (18 °C controls), as were animals that did not contain *DllGAL4* but had gone through the temperature shift to rule out temperature as a causative agent (temperature-shifted controls).

To determine why GAL4 expression in the imaginal disc caused defects in the adult antenna, we examined the antennal imaginal discs after induction of GAL4 expression. *DllGAL4* animals were raised at 18 °C and shifted to 30 °C on day 7 after egg lay (AEL), when they entered the early third larval instar stage. The larvae were kept at 30 °C for a 24-hour period, and examined at different Induction times denoted as I0 and I12 (Fig. 1c), after which they were brought back to 18 °C. The imaginal discs were also observed at multiple points after the temperature shift, over the course of 24 hours, denoted as Recovery times R0-R24 (Fig. 1c).

We confirmed GAL4 expression by marking the *DllGAL4* expression domain using *UAS-EGFP* (Fig. 1d–h). High GAL4 expression causes cell death and can lead to developmental defects, often in a temperature-dependent manner^22,23^. To determine whether the *DllGAL4* expression induced cell death, apoptotic cells were marked by immunostaining for the cleaved form of the effector caspase Dcp-1^24^. 18 °C control discs showed a basal level of apoptosis (Fig. 1i), which was similar to the levels of apoptosis observed in temperature-shifted control discs (Fig. 1i’). Congruent with previous reports^22^, we observed that shifting *DllGAL4* animals to 30 °C resulted in increased apoptosis in the *Dll-*expressing domain, likely due to higher GAL4 expression. Apoptosis progressively increased in discs from I12-R24 (Fig. 1j–m), continuing even after the animals were returned to 18 °C. In the third-instar disc, the apoptosis resulted in altered morphology such that the gap between the peripodial epithelium and the columnar epithelium became more pronounced at R0 (Fig. 1n–q). The gap was the most prominent in the arista region (Fig. 1p–q), which experienced the highest *DllGAL4* expression levels (Fig. 1d). The increased apoptosis was sufficient to cause defects in the arista, and, to a lesser extent, the third antennal segment in the adult animals. The defects were temperature-dependent, as *DllGAL4* animals maintained at 18 °C throughout development almost always had perfectly formed antennae. Six in 156 antennae of animals maintained at 18 °C had very slight deformities of the arista. Even in these antennae the rest of the antennal segments were perfectly formed.

### GAL4-triggered cell death induces a limited damage response

**Figure 2:**
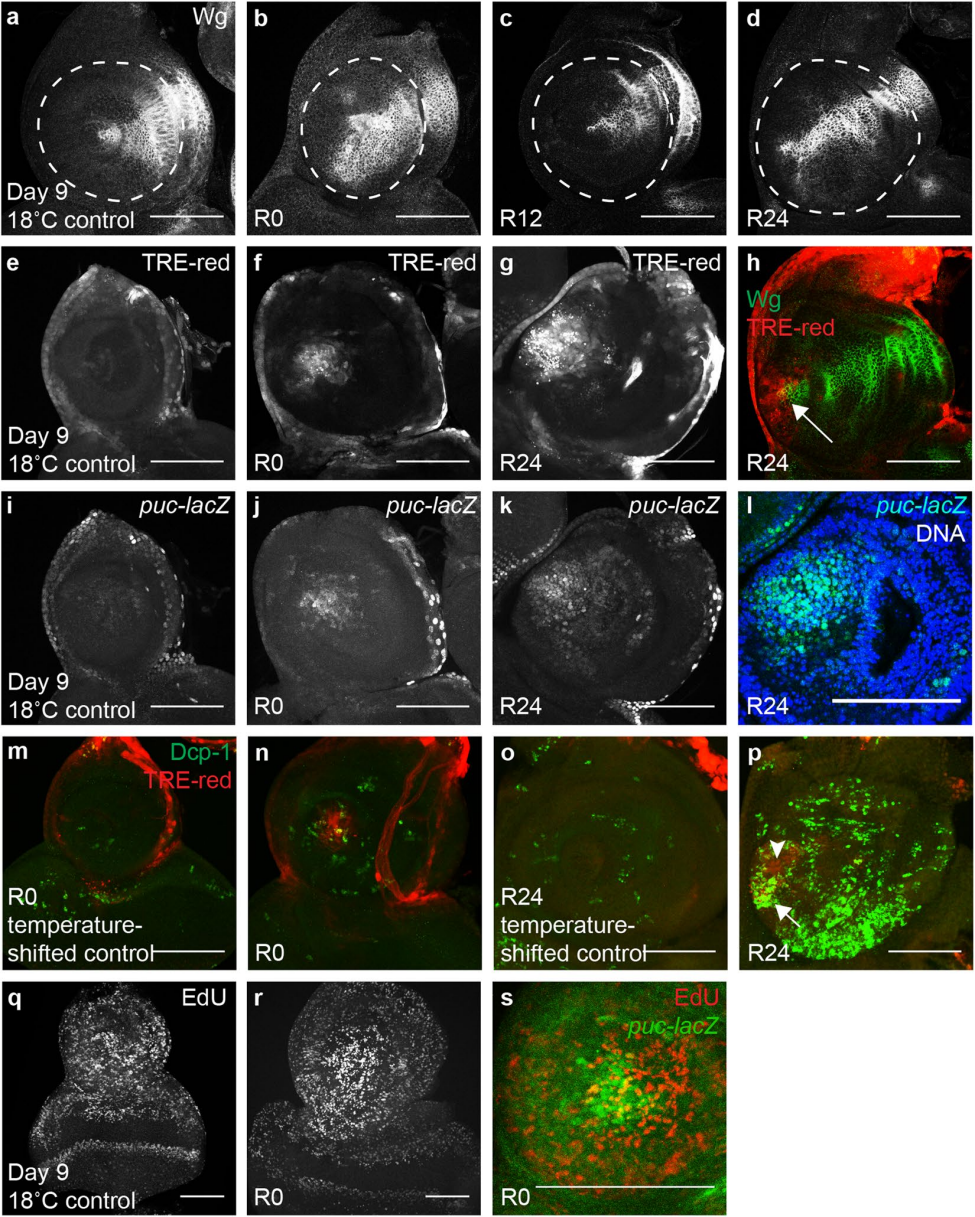
GAL4-triggered cell death elicits a damage response. (**a**–**d**) Anti-Wg immunostaining in a day 9 18 °C control disc (*DllGAL4*/+) (**a**), an R0 disc (**b**), an R12 disc (**c**), and an R24 disc (**d**). Dashed circle in (**a**–**d**) marks approximate *Dll*-expressing region. (**e–g**) TRE-red reporter expression indicating JNK signaling in a day 9 18 °C control disc (**e**), and discs at R0 (**f**) and R24 (**g**). (**h**) Wg (green) co-expression with the TRE-red reporter (red) at R24. Arrow shows JNK signaling co-localized with Wg expression at the center of the disc. (**i**–**k**) *puc-lacZ* expression indicating JNK signaling in a day 9 18 °C control disc (**i**), and discs at R0 (**j**) and R24 (**k**). (**l**) Higher magnification image of (**k**) showing *puc-lacZ* (green) co-localization with epithelial nuclei (blue) marked with TO-PRO-3 at the center of the disc, showing *puc* expression in living cells. (**m**–**p**) Co-expression of Dcp-1 (green) and TRE-red reporter (red) in a temperature-shifted control disc (+; +/*SM6.TM6B*) at R0 (**m**), an (*DllGAL4*/+) R0 disc (**n**), a temperature-shifted control disc at R24 (**o**) and an R24 disc (**p**). (**m** and **o**) TRE-red expression is absent in temperature-shifted control discs. (n and p) Some JNK-expressing cells co-localized with Dcp-1 positive cells (arrow in **p**) while others did not (arrow head in p) showing that not all JNK-expressing cells were apoptotic. (**q** and **r**) EdU incorporation marks cells in S phase in a day 9 18 °C control disc (**q**) and an R0 disc (**r)**. (**s**) *puc-lacZ* (green) co-localization with EdU (red) at R0, showing *puckered* expression in and near proliferating cells. Scale bars are 100 μm.

To determine whether the moderate amount of *DllGAL4*-triggered apoptosis could elicit a tissue-wide damage response, we looked for features characteristic of imaginal disc regeneration or apoptosis-induced proliferation. Wg upregulation near the wound is an early response following damage in the wing and leg imaginal discs^25–27^. In the antennal disc, Wg expression normally occurs in a wedge shape on the dorsal side during early second larval instar, and is maintained in that shape through the second and third instars and into the pupal stages^28^. Wg was present in this characteristic pattern in 18 °C control discs (Fig. 2a). However, after GAL4 activation at higher temperature, the wedge had expanded by R0 such that Wg was present in almost half of the *Dll*-expressing region (Fig. 2b). Wg expression was restored to the characteristic wedge pattern by R12 and remained in the wedge through R24 and into the pupal stage (Fig. 2c–d). Thus, ectopic expression of Wg was observed in response to GAL4-triggered apoptosis.

JNK signaling is triggered after injury and is important for wound healing and regeneration after both surgical and genetic ablation^12,13,27^. Activation of JNK signaling can be monitored through an expression reporter for its transcriptional target *puckered*^29^ or through a transgenic reporter construct controlled by AP-1 binding sites (TRE-red)^30^. Using these reporters, we found that JNK activity was absent in the *Dll* domain in 18 °C control discs (Fig. 2e,i). However, with elevated GAL4 expression at 30 °C, both reporters were expressed at R0 (Fig. 2f,j) and R24 (Fig. 2g,k). The AP-1 reporter was upregulated at the center of the disc (arrow in Fig. 2h). Likewise, *puckered-lacZ* expression showed clear co-localization with epithelial nuclei in the center of the disc, demonstrating *puckered* expression in live cells rather than cellular debris (Fig. 2l). While JNK signaling was upregulated in some cells that were also dying, most of the reporter activity was not associated with apoptosis (Fig. 2m–p).

To examine whether GAL4-triggered apoptosis was inducing proliferation, we visualized proliferating cells by EdU incorporation, which marks the S phase of the cell cycle. EdU incorporation was enriched at the center of the antennal disc in response to GAL4 expression (Fig. 2q–r). Some *puckered-lacZ* expressing cells were also EdU-positive, indicating that JNK was activated in some of the proliferating cells (Fig. 2s).

Myc is important for regenerative growth in wing imaginal discs^26^. To determine whether Myc was elevated during the damage response in the antennal disc, Myc protein levels were observed by immunostaining. Myc was present at low levels throughout the eye-antennal disc in temperature-shifted control discs (Supplemental Fig. 1a). Myc was not upregulated in response to the cell death caused by *DllGAL4* in the antennal imaginal disc (Supplemental Fig. 1b–d). Thus, GAL4-triggered apoptosis in the antennal disc reproduces some elements of a damage response including Wg expression, JNK signaling, and proliferation, but not others, such as elevated Myc expression.

### GAL4-expressing discs experience segment-specific patterning changes

From proximal to distal, the *Drosophila* antenna is made of segments a1, a2, a3 and the arista (ar) (Fig. 1a). We examined gene expression that marks segment identity in the antenna to determine whether GAL4-triggered cell death perturbed disc patterning. For these experiments, we examined the discs at R12 to observe immediate changes in patterning, and we used the temperature-shifted controls to eliminate the possibility of temperature changes causing the patterning defects.

**Figure 3:**
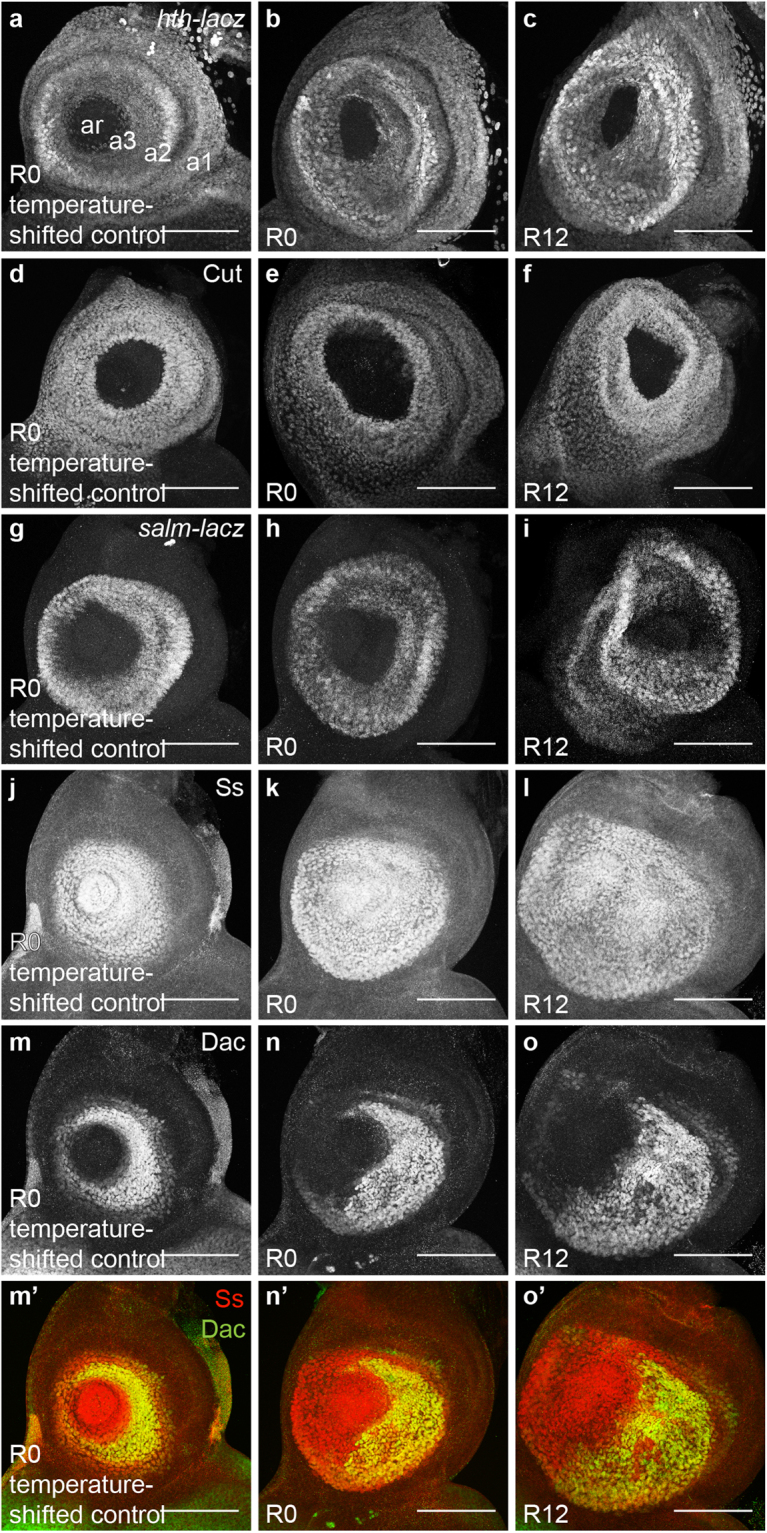
Patterning changes occur in the antennal discs after GAL4-induced cell death. (**a**–**l**) Proximal segment-specific gene expression was not perturbed at R0 and R12. (**a**–**c**) *hth-lacZ* expression in a temperature-shifted control disc (+*; hth-lacZ*/*SM6.TM6B*) at R0 (**a**) and (*DllGAL4*/+*; hth-lacZ*/+) discs at R0 (**b**) and R12 (**c**). (**d**–**f**) Anti-Cut immunostaining in a temperature-shifted control disc (+*;* +/*SM6.TM6B*) at R0 (**d**) and (*DllGAL4*/+) discs at R0 (**e**) and R12 (**f**). (**g**–**i**) *salm-lacZ* expression in a temperature-shifted control disc (*salm-lacZ;* +/*SM6.TM6B*) at R0 (**g**) and (*DllGAL4*/*salm-lacZ*) discs at R0 (**h**) and R12 (**i**). (**j**–**l**) Anti-Spineless (Ss) immunostaining in a temperature-shifted control disc (+*;* +/*SM6.TM6B*) at R0 (**j**) and (*DllGAL4*/+) discs at R0 (**k**) and R12 (**l**). (**m**–**o**) Distal segment-specific gene expression was perturbed as early as R0. Anti-Dachshund (Dac) immunostaining in a temperature-shifted control disc (+*;* +/*SM6.TM6B*) at R0 (**m**) and (*DllGAL4*/+) discs at R0 (**n**) and R12 (**o**). (**m’**–**o’**) Merge of Anti-Ss (**j**–**l**) and Anti-Dac (**m**–**o**) at different time points. Note that Dac expression was perturbed and ventral expression was lost, indicating third segment-specific damage and mispatterning. Scale bars are 100 μm.

In third-instar larvae, *homothorax (hth)* is expressed in a1 and a2, and weakly in a3 (Fig. 3a)^31^. The gene *cut (ct)* is also expressed throughout a1 and a2 (Fig. 3d)^32^. The pattern of these broadly expressed proximal genes remained unchanged at R0 and R12 after *DllGAL4* expression (Fig. 3b,c,e,f), suggesting that the proximal segments, a1 and a2, remained predominantly unaffected, consistent with the adult phenotype. To examine a2 more closely, we observed *spalt major (salm)*, which is expressed in a ring only in a2 (Fig. 3g)^33^. While disc morphology appeared more folded at R12, *salm* expression was not altered at R0 and R12 (Fig. 3h,i), again indicating that a2 was unaffected by *DllGAL4* expression.

To examine more distal antennal segments, we visualized the expression of *spineless (ss)*, which is present in a circular pattern from a3 to ar (Fig. 3j)^34^, and *dachshund (dac)*, which is present in a ring only in a3 (Fig. 3m)^35^. *ss* expression was unchanged and remained within a3-ar at both R0 and R12 (Fig. 3k–l). However, a large region of *dac* expression was lost as early as R0, specifically on the ventral side (Fig. 3n) and remained missing at R12 (Fig. 3o). *dac* expression remained within the *ss* domain, indicating that it did not expand into other antennal segments (Fig. 3m’–o’). Consistent with the adult phenotype, the loss of *dac* expression suggests damage to the third antennal segment.

### GAL4 expression induces antenna-to-eye fate changes

**Figure 4:**
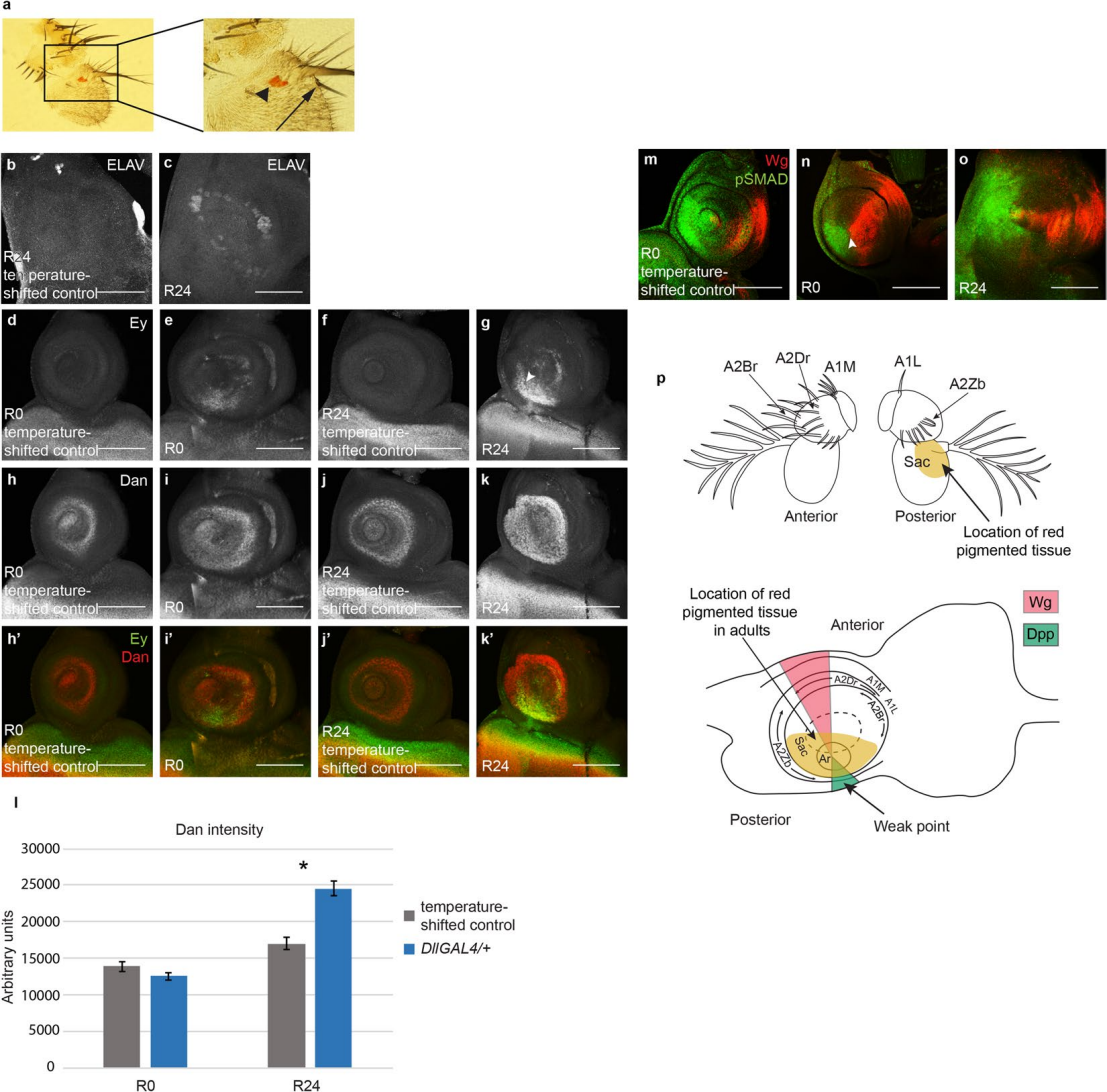
GAL4 expression induces antenna-to-eye fate changes. (**a**) Adult antenna from a *DllGAL4*/+ animal raised at 25 °C. Arrowhead marks the red pigmented tissue in the third antennal segment. Arrow shows the presence of a bracted bristle also on the third antennal segment. (**b**) Anti-ELAV staining was absent in the antennal segments in a temperature-shifted control disc (+*;* +/*SM6.TM6B*) at R24. (**c**) Ectopic ELAV staining occurred in a ring pattern in an (*DllGAL4*/+) R24 disc. (**d**–**g**) Anti-Eyeless (Ey) staining was absent in temperature-shifted control discs at both R0 (**d**) and R24 (**f**). Ey was ectopically expressed as early as R0 (**e**) and increased in area and intensity by R24 (**g**). Note that Ey expression was present near the weak point in the antennal disc, arrowhead in (**g**). (**h**–**k**) Anti-Dan expression in a temperature-shifted control disc at R0 (**h**), an R0 disc (**i**), a temperature-shifted control disc at R24 (**j**) and an R24 disc (**k**). (**h’**–**k’**) Merge of Anti-Ey (**d**–**g**) and Anti-Dan (**h**–**k**). (**d**–**k’**) Images were taken with the same confocal settings to enable comparison of fluorescence levels. (**l**) Graph showing Dan immunofluorescence intensity in temperature-shifted control discs at R0 (n = 16), R0 discs (n = 16), temperature-shifted control discs at R24 (n = 16) and R24 discs (n = 17). *p = 0.000001. (**m**–**o**) Anti-Wg (red) and Anti-pSMAD (green) immunostaining in a temperature-shifted control disc at R0 (**m**) and in discs at R0 (**n**) and R24 (**o**). Arrowhead in (**n**) shows the region of overlap of Wg and pSMAD. (**p**) Fate map of the antennal imaginal disc adapted from Haynie and Bryant^52^. Adult structures of the antenna are labelled showing anterior and posterior views. A1L = antennal segment 1: isolated dorsal lateral bristle, A1M = antennal segment 1: dorsal medial bristles, A2Br = antennal segment 2: large anterior bristles, A2Dr = antennal segment 2: small anterior row bristles, A2Zb = antennal segment 2: posterior tooth bristles, Sac = antennal segment 3: sacculus. Light brown region shows the fate change zone that acquired red pigmented tissue in the adults. The corresponding locations of these structures are labelled on the antennal imaginal disc. Pink and green regions mark Wg and Dpp expression, respectively. Error bars represent SEM. Student’s T-test used for statistical analysis. Scale bars are 100 μm.

The *DllGAL4*-expressing animals that were maintained at 25 °C rather than taken through the 30 °C temperature shift showed more severe damage than shown in Fig. 1b’–b””, as well as outgrowths that appeared similar to leg tissue. Intrigued by these observations, we examined the antennae of animals that were maintained at 25 °C more closely. We found evidence of fate changes in these antennae, such as the presence of bracted bristles that are normally only found on the distal segments of the legs^36^ and the proximal costa of the wings^37^ (Fig. 4a). Antenna-to-leg and antenna-to-wing transdetermination events have been observed before^38^. Incredibly, we also found what appeared to be pigmented eye tissue in some of the affected antennae (Fig. 4a), a fate change that has not been previously reported as occurring after tissue damage. We also observed the antenna-to-eye fate change in antennae of animals that underwent the 30 °C temperature shift. These fate changes were dependent on GAL4 expression, as they were completely suppressed by expression of GAL80^39^ in the *DllGAL4* animals maintained at 25 °C (0 in 488 antennae showed any defects, 2 independent experiments) (Supplemental Table 1).

To confirm that the red-pigmented tissue in the antenna was indeed aberrant eye tissue, we examined expression of the proneural marker ELAV in the antennal discs of animals that underwent the 30 °C temperature shift to identify photoreceptor precursors. ELAV immunostaining was never observed in temperature-shifted control discs (Fig. 4b), while aberrant ELAV expression was observed in a ring in GAL4-expressing discs at R24 (Fig. 4c). We also examined Eyeless (Ey) expression, as *ey* is an eye selector gene and can ectopically induce eye tissue^40^. Ey immunostaining was indeed observed as early as R0, and expression increased in area and intensity by R24 (Fig. 4d–g). Interestingly, Ey was expressed in an asymmetric ring, with much wider and intense expression on the ventral posterior side, which may coincide with the weak point (Fig. 4p). The presence of Ey in this particular domain could explain why red-pigmented tissue was only observed on the posterior side of the third antennal segment and not in a ring (Fig. 4a). Ey expression could be conferring competence to the cells in this domain, enabling ELAV-expressing cells to recruit pigment cells and other eye cell types to form rudimentary eyes, while ELAV-expressing cells in other portions of the imaginal disc either did not survive to adulthood, did not remain neuronal precursors, or were not visible due to lack of red pigment cells.

The genes *distal antenna (dan)* and *distal antenna related (danr)* are effector genes that are involved in antennal fate specification^41^. However, over-expression of *dan* and *danr* induces the formation of eye tissue in the third antennal segment^42,43^. *dan* expression is normally present in the antennal disc from a3-ar (Fig. 4h,j). Interestingly, expression of *DllGAL4* caused upregulation of *dan* expression in R24 discs (Fig. 4h–l), consistent with a change to eye fate. These data suggest that GAL4 expression can cause gene expression changes in the antennal disc, resulting in a subset of cells adopting eye fate.

It was unclear whether the disc region that produced the visible eye tissue in the adult constituted a classic weak point as defined in the transdetermination literature. Interaction between Wg and Dpp signaling is able to induce transdetermination at the weak points, and the weak point in the antennal disc is the region of Dpp expression on the ventral side of the disc^11^. To determine whether any ectopic interaction of Wg and Dpp was occurring in the *DllGAL4*-expressing discs, we examined Wg expression and Dpp signaling, as marked by pSMAD immunostaining. Wg and pSMAD are present in two opposing wedges that overlap at the center of a temperature-shifted control antennal disc (Fig. 4m)^28^. Upon *DllGAL4* expression, the Wg expression domain expanded radially right up to the Dpp signaling domain at R0 (Fig. 4n), and a very slight overlap of Wg and pSMAD was seen where the two domains met (arrowhead in Fig. 4n). Subsequently, the Wg and Dpp domains separated^44^, and the Wg expression domain was restored to the characteristic wedge pattern by R12 (Fig. 2e). By R24, Dpp signaling had diminished and no overlap with Wg was observed except at the center (Fig. 4o). Importantly, the observed region of ectopic expression of Ey includes the portion of the disc in which Wg and Dpp overlap at R0.

### Distinguishing between transdetermination and homeosis as the cause for the GAL4-induced fate changes

There are several possible ways in which the *DllGAL4* line could be causing fate changes. The *DllGAL4* enhancer trap is also a mutant for the *Dll* gene. Thus, the *Dll* mutation alone could cause homeotic fate changes in the antennae, although this possibility is unlikely as GAL80 suppressed the fate changes, indicating a requirement for GAL4 activity. Alternatively, GAL4 could perturb gene expression, leading to fate changes, or the cell death and damage response induced by the GAL4 expression could cause the fate changes. Finally, the combination of GAL4 expression in the *Dll* mutant background could be causing the aberrant cell plasticity.

To rule out the possibility that the fate changes were solely a result of a homeotic mutation in the *Dll* locus, we examined three other *Dll* mutants: *Dll*^*5*^ ^45^, *Dll*^01092^ ^46^ and *Dll*^*9*^ ^47^. None of these mutant alleles showed any red-pigmented tissue in the adult antennae when heterozygous (0 in 206, 0 in 224 and 0 in 258 antennae, respectively) (Supplemental Table 1). These results indicate that homeosis alone is unable to produce the antenna-to-eye fate change in *Dll* heterozygotes.

**Figure 5:**
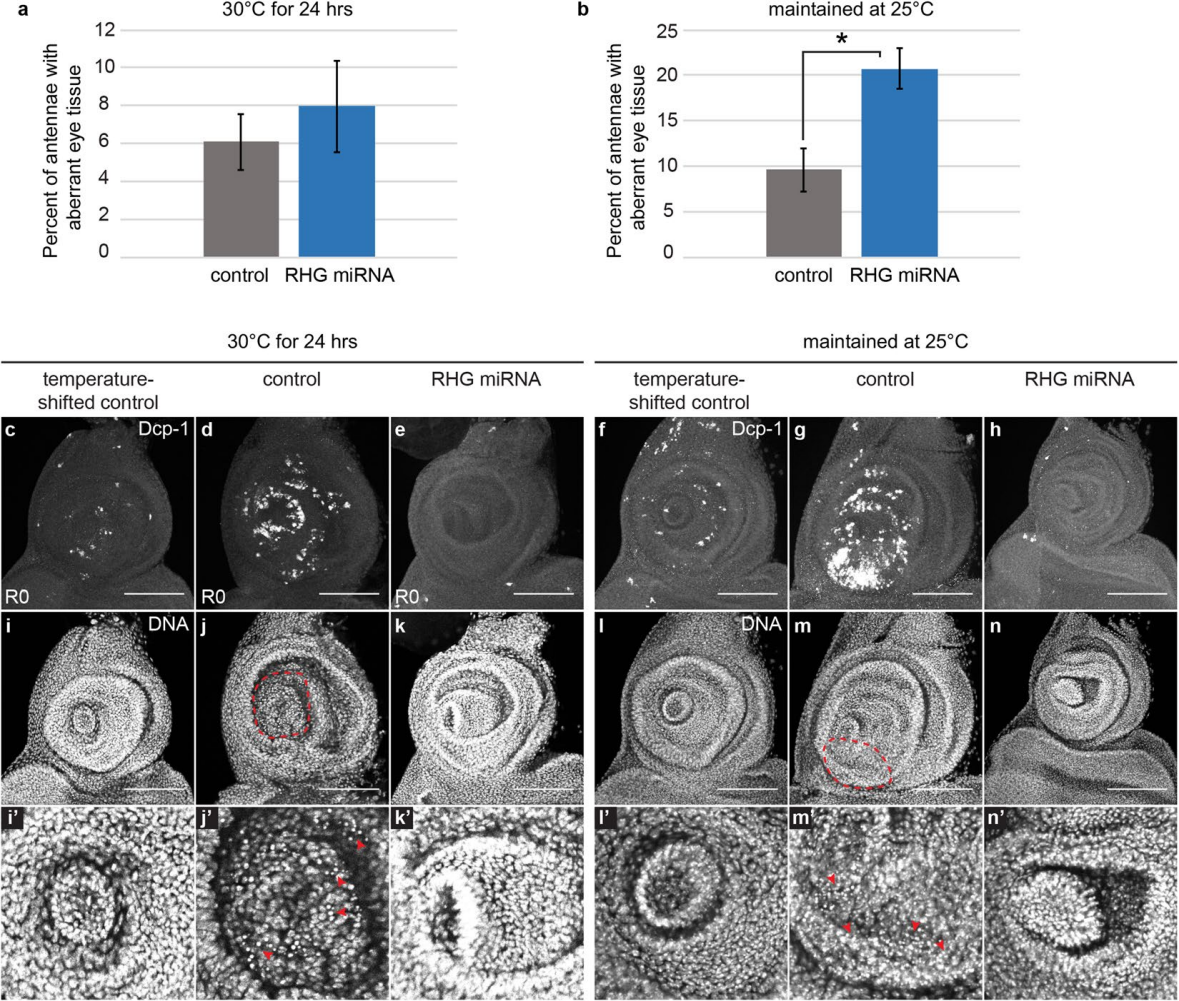
Regulation of fate changes. (**a** and **b**) Frequency of red-pigmented tissue in adult antennae of animals expressing miRNAs targeting *reaper, hid* and *grim* (RHG miRNA) under *DllGAL4* control, compared to controls. (**a**) Animals underwent the 30 °C temperature shift. 4 independent experiments, control (*DllGAL4*/+) n = 258 antennae, RHG miRNA (*DllGAL4*/*UASRHG miRNA*) n = 116 antennae. (**b**) Animals were maintained at 25 °C. 3 independent experiments, control (*DllGAL4*/+) n = 476 antennae, RHG miRNA (*DllGAL4*/*UASRHG miRNA*) n = 294 antennae. *p = 0.02. (**c**–**e** and **i**–**k’**) Examination of cell death by cleaved Dcp-1 immunostaining and pyknotic nuclei identified by DAPI-marked DNA in animals that underwent the 30 °C temperature shift. (**i’**–**k’**) Magnified view of (**i**–**k**). (**c** and **i**–**i’**) Cleaved Dcp-1 and DAPI in a temperature-shifted control disc (+*;* +/*SM6.TM6B*) at R0. (**d** and **j–j’**) Cleaved Dcp-1 and DAPI in a *DllGAL4*/+ disc at R0. Red outlined area (**j**) and arrowheads (**j’**) mark location of pyknotic nuclei indicating dying cells. (**e** and **k**–**k’**) Cleaved Dcp-1 and DAPI in a *DllGAL4* disc also expressing the RHG miRNA (*DllGAL4*/*UASRHG miRNA*) at R0. Note the absence of dying cells as detected by molecular marker or by morphology. (**f**–**h** and **l**–**n’**) Examination of cell death by cleaved Dcp-1 immunostaining and TO-PRO3-marked DNA for observing pyknotic nuclei in animals that were maintained at 25 °C. All discs were dissected from third-instar larvae. (**l’**–**n’**) Magnified view of (**l**–**n**). (**f** and **l**–**l’**) Cleaved Dcp-1 and TO-PRO-3 in a temperature-shifted control disc. (**g** and **m**–**m’**) Cleaved Dcp-1 and TO-PRO-3 in a *DllGAL4*/+ disc. Red outlined area (**m**) and arrowheads (**m’**) mark location of pyknotic nuclei. (**h** and **n**–**n’**) Cleaved Dcp-1 and TO-PRO-3 in a *DllGAL4* disc also expressing the RHG miRNA (*DllGAL4*/*UASRHG miRNA*). Note the absence of dying cells as detected by molecular marker or by morphology. Error bars represent SEM. Student’s T-test used for statistical analysis. Scale bars are 100 μm.

While we have shown that GAL4 activity is required for the antenna-to-eye fate changes in the *DllGAL4*/+ animals, it was unclear whether the fate changes were due to direct activity of GAL4 on gene expression, cellular stress caused by high expression of an exogenous protein, or a transdetermination event induced by the apoptotic cells. To determine whether the fate changes were caused by the *DllGAL4*-triggered apoptosis, we blocked caspase-mediated cell death by expressing a miRNA targeting *reaper, hid* and *grim*^48^ under *DllGAL4* control either for a 24-hour period at 30 °C or by continuously maintaining the animals at 25 °C. Controls were *DllGAL4* animals not expressing the miRNA. Strikingly, in both these experiments, the frequency of fate change was not decreased as would be expected if apoptosis in nearby cells or the damage response were the cause of the fate change (Fig. 5a–b). On the contrary, the fate change frequency was significantly increased upon block of apoptosis in animals always maintained at 25 °C (Fig. 5b). Absence of apoptotic cells was confirmed by immunostaining for the cleaved form of the effector caspase Dcp-1 (Fig. 5c–h). We also examined the *rpr, hid, grim* miRNA-expressing imaginal discs for pyknotic nuclei to rule out other forms of cell death. Our examination did not show any clear evidence of dying cells (Fig. 5i–n). These results suggest that high *DllGAL4* expression might result in aberrant signaling or cellular stress independent of cell death, making this fate change different from classical transdetermination events. Interestingly, these results also suggest that caspase activation and/or apoptosis play important roles in restricting the frequency of fate changes in the antenna.

If the fate changes were a result of GAL4-induced aberrant gene expression or cellular stress, GAL4 driven by another promoter in the same domain should also produce the same phenotype. To assess this hypothesis, we used a transgenic *ssGAL4*, as *ss* is expressed from a3 to ar (Fig. 3j). This GAL4 transgene does not affect the endogenous *ss* gene and hence is not a mutant. We confirmed GAL4 expression by marking the *ssGAL4* expression domain using *UAS-EGFP* in heterozygous *ssGAL4* animals (Supplemental Fig. 2b). *ssGAL4* expression was slightly lower than *DllGAL4* expression (Supplemental Fig. 2a–c). Adult antennae of homozygous *ssGAL4* animals showed defects similar to *DllGAL4* animals, specifically altered morphology in the arista and the third antennal segment (Supplemental Fig. 2d–d””). Compared to wild-type controls, homozygous *ssGAL4* animals showed much higher levels of cell death, which could explain the altered morphology of the adults (Supplemental Fig. 2e–f). While heterozygous *ssGAL4* activity increased with temperature as observed by GFP expression (Supplemental Fig. 2g,j), there was no apparent increase in cell death (Supplemental Fig. 2h,k). Interestingly, we did not observe any red-pigmented tissue in adult antennae of homozygous *ssGAL4* animals maintained at 25 °C (0 in 334 antennae) (Supplemental Table 1).

We also examined homozygous *ssGAL4* antennal discs for Ey and ELAV expression to see if the cells were undergoing fate changes that were not apparent at the adult stage. No Ey or ELAV expression was observed in the antennal portion of the wild-type control discs (Supplemental Fig. 2m). By contrast, while ELAV staining was not observed, Ey expression was seen in homozygous *ssGAL4* discs (Supplemental Fig. 2n). However, the Ey expression was not present near the weak point as observed for the *DllGAL4* discs. These results suggest that low levels of Ey may be expressed in the homozygous *ssGAL4* discs, but it not enough and not in the right place to lead to fate changes observable in the adult antenna.

It is possible that GAL4 alone may not be sufficient to drive fate changes unless it is expressed in a *Dll* heterozygous mutant background. To test this hypothesis, we examined adult antennae of *ssGAL4* animals crossed to multiple *Dll* mutants. However, we did not detect any antennae with red-pigmented tissue: 0 in 290 antennae for *Dll*^01092^/+; *ssGAL4*/+ animals, 0 in 302 antennae for *Dll*^*9*^/+; *ssGAL4*/+ animals, and 0 in 470 antennae for *Dll*^*5*^/+*; ssGAL4*/+ animals (Supplemental Table 1). *Dll*^*5*^/+ animals showed antenna-to-leg transformations, and the frequency of these transformation events increased from 5.2% in *Dll*^*5*^/+ animals to 19.4% in *Dll*^*5*^/+; *ssGAL4*/+ animals (Supplemental Table 2). Thus, while *ssGAL4* expression can enhance the homeosis observed in *Dll*^*5*^/+, it does not induce the eye fates observed in the *DllGAL*4 line. We also examined adult antennae of homozygous *ssGAL4* animals crossed to multiple *Dll* mutants. Again, we did not observe any antennae with red-pigmented tissue: 0 in 260 antennae for *Dll*^*9*^/+*; ssGAL4*/*ssGAL4* animals and 0 in 232 antennae in *Dll*^*5*^/+*; ssGAL4*/*ssGAL*4 animals (Supplemental Table 1). However, we did observe necrotic tissue that was present in a region similar to where the red-pigmented tissue was observed (Supplemental Fig. 2o–o’). The necrotic tissue appeared with a frequency of 3.07% in *Dll*^*9*^/+*; ssGAL4*/*ssGAL4* animals and 1.29% in *Dll*^*5*^/+*; ssGAL4*/*ssGAL4* animals (Supplemental Table 3). These data support our hypothesis that fate changes may be initiated in *ssGAL4* animals but do not go through the full differentiation process to result in red-pigmented tissue in the adults.

Heterozygous *DllGAL4* animals have slightly higher GAL4 activity as compared to heterozygous *ssGAL4* animals (Supplemental Fig. 2a–c). They also have much lower levels of Dll protein as compared to controls, but Dll is still expressed in the correct domain (Supplemental Fig. 3a–c). To test whether the frequency of fate changes in the *Dll* heterozygous background depends on the dose of GAL4 activity, we combined the *DllGAL4* and *ssGAL4* constructs and quantified the frequency of pigmented eye tissue in the antennae. Surprisingly, the combination of the two GAL4s resulted in complete loss of pigmented eye tissue in the antennae (0 in 442 antennae for *DllGAL4*/+*; ssGAL4*/+ animals, 2 independent experiments) (Supplemental Table 1). The adult antennae of these animals still showed defects in the third antennal segment and the arista. We examined Dcp-1 levels in *DllGAL4* and *DllGAL4*/+*; ssGAL4*/+ animals to investigate the differences in levels of cell death in these two genotypes. We found that *DllGAL4*/+*; ssGAL4*/+ antennal discs showed higher levels of cell death as compared to *DllGAL4* discs (Supplemental Fig. 3d–e). Thus, it is possible that high levels of cell death caused by the extremely high GAL4 activity led to loss of the cells that would have changed fate. This finding is consistent with the observed increase in frequency of fate changes when cell death was blocked (Fig. 5), and is also consistent with a recent study describing notum-to-wing transformations where increasing apoptosis suppressed the formation of ectopic wings^49^.

To rule out the possibility that the fate change may be caused by the background of the *DllGAL4* line (+*; DllGAL4*/*CyO*) that is maintained in the Bloomington Drosophila Stock Center, possibly due to an enhancer mutation that has arisen recently in the stock, we obtained *y, w, hsFLP; DllGAL4*/*CyO* from a different source, further altered the background by outcrossing to a *w*^1118^ line for two generations, and confirmed the antenna-to-eye fate change in that line as well. We were also unable to detect the presence of any UAS elements by PCR in our *DllGAL4* stock, ruling out the possibility of contamination with an unknown transgene that induces overexpression of eye fate genes under *DllGAL4* control. While it remains possible that an enhancer is present that is tightly linked to the *DllGAL4*, we propose that some unique combination of the strength of GAL4 expression and severity of the *Dll* mutation in the *DllGAL4* line enable this unprecedented plasticity in the antennal disc.

## Discussion

Given the results presented here, we propose a model in which the high GAL4 expression in the *DllGAL4* mutant line perturbs normal gene expression and induces cell fate changes in the antennal imaginal disc. We do not yet know the mechanism through which GAL4 induces the fate change to eye tissue, but it is independent of GAL4-triggered apoptosis. Regardless, this study highlights the functional impact that GAL4 expression can have on *Drosophila* tissues that are being manipulated genetically, and researchers must be aware of the perturbations that GAL4 expression may cause. Interestingly, *DllGAL4* is usually balanced over a *CyO* chromosome, which we found suppresses the frequency of the fate change (Supplemental Table 1). This balancer-induced suppression may explain why this fate change has not been previously reported. Here the fate change appears to be specific to the *DllGAL4* line, in which high GAL4 expression, possibly combined with this particular *Dll* mutation, produces the phenotype in this unique context. It is possible that in other contexts, GAL4 expression may cause perturbations that lead to different kinds of developmental phenotypes.

The genetic utility of the GAL4/UAS system has been of immense importance in *Drosophila* and has been employed to control gene expression in specific spatial domains^50^. Since GAL4 is a yeast transcription factor, it is believed to have little or no effect on the endogenous promoters/enhancers in *Drosophila*. However, there have been recent reports where high GAL4 expression causes cell death and can lead to developmental defects. Increased GAL4 dosage in a subset of neurons affects neuronal physiology and behavior in the animals, which was associated with apoptotic neuronal loss in the GAL4-expressing neurons. Introduction of either chaperone proteins or inhibition of cell death, but not expression of GAL80, was able to rescue these defects, supporting the hypothesis that increased GAL4 dosage in the neurons causes protein misfolding, resulting in protein aggregates leading to cellular stress and cell death^23^. *GMR-GAL4* in the *Drosophila* eye induces apoptosis and leads to the formation of irregular ommatidial arrays in a dosage- and temperature-dependent manner^22^. In this case, the GAL4-associated defects can be rescued by constitutive expression of GAL80, suggesting that GAL4 may be activating the misexpression of certain endogenous genes that leads to developmental defects^23^. These findings suggest that the effects of high GAL4 expression can, in some cases, be independent of cell death. Therefore, because high GAL4 expression can stress cells and change them, care should be taken, and proper controls included when designing *Drosophila* experiments.

We found that neither *Dll* mutations nor GAL4 expression on their own is able to transform antennal tissue to eye tissue such that red pigment is visible in the adult antenna. We found that the combination of *Dll* mutations with *ssGAL4* expression was also unable to cause the transformation. However, we did observe ectopic Ey expression in *ssGAL4* animals, suggesting perturbations in the normal development of these cells. Finally, we observed that GAL80 completely rescued the fate transformation phenotype. From this result, we can conclude that GAL4 activity is required for the antenna-to-eye transformation.

Sporadic fate changes in a small part of an imaginal disc are usually a consequence of transdetermination, which could be induced by damage or by ectopic induction of Wg signaling. Another way that fate changes can be induced is through homeotic mutations. This particular antenna-to-eye fate change may be caused by a combination of both processes, as we observed ectopic Wg expression, but the fate change was specific to the mutant *DllGAL4* background. However, neither transdetermination nor homeosis on its own is able to explain the phenotype, since cell death does not contribute to this fate change, and other mutations in the *Dll* locus do not lead to eye tissue formation in the antennae. Furthermore, overexpression of Wg using *dppGAL4* or *patchedGAL4* cannot by itself cause antenna-to-eye transformations^18^. It is possible that high GAL4 activity perturbs the expression of genes in the eye-specification network such as *eyeless* and *dan*, and the *Distal-less* heterozygous mutant background sensitizes the antennae to these perturbations, allowing the fate change to eye.

The location of the transformed tissue is broadly consistent with previous literature. Classical transdetermination studies have shown that presumptive cells of the third antennal segment and the arista can change fate to other organs. The second antennal segment, which proliferates at a lower rate, rarely ever forms transdetermined structures^38^. Our results show that the *DllGAL4*-induced fate change occurred in the third antennal segment. While the classical tissue damage work by Gehring identified antennal disc transdetermination to portions of the eye disc that form head structures, it did not identify transdetermination to eye tissue marked by red pigment or photoreceptors^16,38,51^. To our knowledge, this study is the first report of antennal tissue transforming to pigmented eye tissue without the use of hox gene mutants or ectopic expression of eye fate genes.

The weak point in the antennal disc has been identified as the region of Dpp expression on the ventral side of the disc^11^ (Fig. 4h). In our experiments, the ectopic eye tissue was always seen on the posterior side of the third antennal segment in the adult animals. Using the fate map of the antennal disc developed by Haynie and Bryant, we determined that the transformation zone extends radially from the arista/third segment border to some distance in the posterior half of the third antennal segment^52^ (Fig. 4p). While we observed ELAV-expressing cells present in a ring pattern in the antennal imaginal disc, Ey was only primarily expressed in a region overlapping the antennal weak point. It is possible that the ELAV-expressing cells outside of the weak spot are in tissue that is not competent to form pigment cells. We also observed loss of Dac expression in this zone in the antennal imaginal disc (Fig. 3n–o). Interestingly, Dac downregulation is necessary but not sufficient for leg-to-wing transdetermination^53^. Thus, the observed loss of Dac may also be partly defining the transformation-competent zone in the antenna.

Our findings emphasize the need for further work to understand the circumstances under which fate changes can be induced. The antenna-to-eye fate change that we report here differs from previously reported transformations in that it is not induced by damage or homeotic mutations. We conclude that it is possible for determined cell fates to change in the absence of tissue damage but in the presence of other forms of cellular stress caused by high GAL4 activity, and that selector gene mutations sensitize the tissue to these forms of transformations.

## Materials and Methods

### GAL4-induced damage protocol

*DllGAL4* females were crossed to *w*^1118^ males or males harboring specific mutations or transgenes. Eggs were collected at room temperature in 5-hour intervals on grape juice plates and subsequently maintained at 18 °C or 25 °C. 50 newly hatched larvae were collected into each vial 48 or 24 hours later, respectively. The vials contained food prepared according to the standard medium recipe provided by the Bloomington Drosophila Stock Center, which was churned and supplemented with yeast paste. Vials containing larvae that had been maintained at 18 °C were placed in a 30 °C circulating water bath on day 7 after egg lay. The vials were kept at 30 °C for a 24-hour period after which they were cooled in an ice-water bath for 60 seconds and returned to 18 °C. In some noted experiments, larvae were maintained constantly at 25 °C. Controls were always maintained at 18 °C. Temperature-shifted controls were the siblings of damaged animals, with the genotype *w*^1118^*;* +/*SM6.TM6B*, which were taken through the 30 °C temperature shift or were maintained at 25 °C.

### Fly stocks

The following *Drosophila* stocks were used: *Dll*^*md23*^ (called *DllGAL4* in the text)^20^, *Dll*^*5*^^45^, *Dll*^01092 ^^46^*, Dll*^*9*^^47^*, w*^1118^*; P{GMR14C05-GAL4}attP2* (called *ssGAL4* in the text)^54^*, w*^1118^ (Wild-type)^55^, *Cyo, tubGAL80*^39^, *UASEGFP*^56^, the AP-1 reporter TRE-red^30^ (a gift from Dirk Bohmann, University of Rochester), *hth*^05745 ^^57^, *salm*^03602 ^^57^, *puc*^*E69 *^^29^, *UASRHG miRNA*^48^ (a gift from Sarah Siegrist, University of Virginia) and *y, w, hsFLP; DllGAL4*/*CyO* (obtained from Xin Li, University of Illinois Urbana-Champaign). All fly stocks are available from the Bloomington Drosophila Stock Center unless stated otherwise.

### Immunohistochemistry

Immunostaining was carried out as previously described^26^. Primary antibodies were rabbit anti-Cleaved *Drosophila* Dcp-1 (1:250) (Cell Signaling), mouse anti-Wingless (1:100) (The Developmental Studies Hybridoma Bank [DSHB]), rabbit anti-dMyc (1:500) (Santa Cruz Biotechnologies), mouse anti-βgal (1:100) (DSHB), mouse anti-Cut (1:10) (DSHB), mouse anti-Dac (1:5) (DSHB), guinea pig anti-Spineless (1:200) (a gift from Michael Kim, University of Miami), rat anti-ELAV (1:30) (DSHB), mouse anti-Ey (1:100) (DSHB), rat anti-Dan^41^ (1:200) (obtained from Xin Li, University of Illinois Urbana-Champaign), rabbit anti-phospho-Mad (1:100) (Cell Signaling), rabbit anti-Distal-less (1:200) (a gift from Sean Carroll, University of Wisconsin-Madison). The Developmental Studies Hybridoma Bank (DSHB) was created by the NICHD of the NIH and is maintained at the University of Iowa, Department of Biology, Iowa City, IA 52242.

Secondary antibodies were AlexaFluor probes (1:1000) (Life Technologies). DNA was marked using TO-PRO3 (1:500) (Life Technologies) or DAPI (1:5000 of 0.5 mg/mL stock) (Sigma). Discs were mounted in Vectashield mounting medium (Vector Laboratories).

EdU labeling was carried out as previously described^58^ using the Click-It EdU kit (Life Technologies). Tissue was incubated in EdU for 20 minutes at room temperature.

Discs were imaged on a Zeiss LSM 510 or a Zeiss LSM 700 confocal microscope. Parameters for imaging as well as brightness and contrast settings were set to optimize for expression and location rather than for quantitative comparison unless stated specifically. Images were processed using ZEN lite (Zeiss), ImageJ (NIH) and Photoshop (Adobe).

### Adult antenna microscopy

Adult antennae were mounted in Gary’s Magic Mount (Canada balsam [Sigma] dissolved in methyl salicylate [Sigma]). Images were taken with an Olympus SZX10 microscope and a Leica DM RXA2 microscope with an Olympus DP21 camera using the CellSens Dimension software (Olympus).

### Data availability

The data and images generated during and/or analysed during the current study are available from the corresponding author on reasonable request.

## Electronic supplementary material

Supplementary Information

## References

[1] Lawrence PA, Morata G (1994). Homeobox genes: their function in Drosophila segmentation and pattern formation. Cell.

[2] Simcox AA, Sang JH (1983). When does determination occur in Drosophila embryos?. Dev. Biol..

[3] Meise M, Janning W (1993). Cell lineage of larval and imaginal thoracic anlagen cells of Drosophila melanogaster, as revealed by single-cell transplantations. Development.

[4] Fuse N, Hirose S, Hayashi S (1996). Determination of wing cell fate by the escargot and snail genes in Drosophila. Development.

[5] Kauffman, S. A. Pattern formation in the Drosophila embryo. *Philos. Trans. R. Soc. Lond. B. Biol. Sci*. 567–594 (1981). 10.1098/rstb.1981.0161 6117912

[6] Hadorn E (1963). Differenzierungsleistungen wiederholt fragmentierter Teilstücke männlicher Genitalscheiben von Drosophila melanogaster nach Kultur *in vivo*. Dev. Biol..

[7] Schubiger G (1971). Regeneration, duplication and transdetermination in fragments of the leg disc of Drosophila melanogaster. Dev. Biol..

[8] Hadorn E (1965). Problems of determination and transdetermination. Brookhaven Symp. Biol..

[9] Maves L, Schubiger G (1995). Wingless induces transdetermination in developing Drosophila imaginal discs. Development.

[10] Maves L, Schubiger G (1998). A molecular basis for transdetermination in Drosophila imaginal discs: interactions between wingless and decapentaplegic signaling. Development.

[11] Maves L, Schubiger G (2003). Transdetermination in Drosophila imaginal discs: a model for understanding pluripotency and selector gene maintenance. Curr. Opin. Genet. Dev..

[12] Bosch M, Serras F, Martín-Blanco E, Baguñà J (2005). JNK signaling pathway required for wound healing in regenerating Drosophila wing imaginal discs. Dev. Biol..

[13] Bergantinos C, Corominas M, Serras F (2010). Cell death-induced regeneration in wing imaginal discs requires JNK signalling. Development.

[14] Lee N, Maurange C, Ringrose L, Paro R (2005). Suppression of Polycomb group proteins by JNK signalling induces transdetermination in Drosophila imaginal discs. Nature.

[15] Karlsson J (1979). A major difference bwteen transdetermination and homeosis. Nature.

[16] Schmid H (1985). Transdetermination in the homeotic eye-antenna imaginal disc of Drosophila melanogaster. Dev. Biol..

[17] Kauffman SA (1973). Control Circuits for Determination and Transdetermination. Science.

[18] Johnston LA, Schubiger G (1996). Ectopic expression of wingless in imaginal discs interferes with decapentaplegic expression and alters cell determination. Development.

[19] McClure KD, Schubiger G (2007). Transdetermination: Drosophila imaginal disc cells exhibit stem cell-like potency. Int. J. Biochem. Cell Biol..

[20] Calleja M, Moreno E, Pelaz S, Morata G (1996). Visualization of Gene Expression in Living Adult Drosophila. Science.

[21] Brand, A. H., Manoukian, A. S. & Perrimon, N. Chapter 33 Ectopic Expression in Drosophila. in *Methods in Cell Biology* (eds Goldstein, L. S. B. & Fyrberg, E. A.) **44**, 635–654 (Academic Press, 1994). 10.1016/s0091-679x(08)60936-x 7707973

[22] Kramer JM, Staveley BE (2003). GAL4 causes developmental defects and apoptosis when expressed in the developing eye of Drosophila melanogaster. Genet Mol Res.

[23] Rezával C, Werbajh S, Ceriani MF (2007). Neuronal death in Drosophila triggered by GAL4 accumulation: Neuronal death by protein overload. Eur. J. Neurosci..

[24] Song Z, McCall K, Steller H (1997). DCP-1, a Drosophila Cell Death Protease Essential for Development. Science.

[25] Schubiger M, Sustar A, Schubiger G (2010). Regeneration and transdetermination: The role of wingless and its regulation. Dev. Biol..

[26] Smith-Bolton RK, Worley MI, Kanda H, Hariharan IK (2009). Regenerative Growth in Drosophila Imaginal Discs Is Regulated by Wingless and Myc. Dev. Cell.

[27] Ryoo HD, Gorenc T, Steller H (2004). Apoptotic Cells Can Induce Compensatory Cell Proliferation through the JNK and the Wingless Signaling Pathways. Dev. Cell.

[28] Lebreton G, Faucher C, Cribbs DL, Benassayag C (2008). Timing of Wingless signalling distinguishes maxillary and antennal identities in Drosophila melanogaster. Development.

[29] Martín-Blanco E (1998). Puckered encodes a phosphatase that mediates a feedback loop regulating JNK activity during dorsal closure in Drosophila. Genes Dev..

[30] Chatterjee N, Bohmann D (2012). A Versatile ΦC31 Based Reporter System for Measuring AP-1 and Nrf2 Signaling in Drosophila and in Tissue Culture. PloS One.

[31] Dong PD, Chu J, Panganiban G (2000). Coexpression of the homeobox genes Distal-less and homothorax determines Drosophila antennal identity. Development.

[32] Dong PS, Dicks JS, Panganiban G (2002). Distal-less and homothorax regulate multiple targets to pattern the Drosophila antenna. Development.

[33] Barrio R, de Celis JF, Bolshakov S, Kafatos FC (1999). Identification of Regulatory Regions Driving the Expression of the Drosophila spalt Complex at Different Developmental Stages. Dev. Biol..

[34] Duncan DM, Burgess EA, Duncan I (1998). Control of distal antennal identity and tarsal development inDrosophila by spineless–aristapedia, a homolog of the mammalian dioxin receptor. Genes Dev..

[35] Dong PS, Chu J, Panganiban G (2001). Proximodistal domain specification and interactions in developing Drosophila appendages. Development.

[36] Hannah-Alava A (1958). Morphology and chaetotaxy of the legs of Drosophila melanogaster. J. Morphol..

[37] Bryant PJ (1975). Pattern formation in the imaginal wing disc of Drosophila melanogaster: fate map, regeneration and duplication. J. Exp. Zool..

[38] Gehring W (1967). Clonal analysis of determination dynamics in cultures of imaginal disks in Drosophila melanogaster. Dev. Biol..

[39] Vef O, Cleppien D, Löffler T, Altenhein B, Technau GM (2006). A new strategy for efficient *in vivo* screening of mutagenized Drosophila embryos. Dev. Genes Evol..

[40] Halder, G., Callaerts, P., Gehring, W. J. & others. Induction of ectopic eyes by targeted expression of the eyeless gene in Drosophila. *Sci.-N. Y. Then Wash*.- 1788–1788 (1995). 10.1126/science.7892602 7892602

[41] Emerald BS (2003). Distal antenna and distal antenna related encode nuclear proteins containing pipsqueak motifs involved in antenna development in Drosophila. Development.

[42] Suzanne M, Estella C, Calleja M, Sánchez-Herrero E (2003). The hernandez and fernandez genes of Drosophila specify eye and antenna. Dev. Biol..

[43] Curtiss J, Burnett M, Mlodzik M (2007). distal antenna and distal antenna-related function in the retinal determination network during eye development in Drosophila. Dev. Biol..

[44] Domínguez M, Casares F (2005). Organ specification-growth control connection: New in- *sights* from the Drosophila eye-antennal disc: Drosophila Eye-Antennal Disc. Dev. Dyn..

[45] Sunkel CE, Whittle JRS (1987). Brista: a gene involved in the specification and differentiation of distal cephalic and thoracic structures in Drosophila melanogaster. Dev. Genes Evol..

[46] Goto S, Hayashi S (1997). Specification of the embryonic limb primordium by graded activity of Decapentaplegic. Development.

[47] Sato T (1984). A new homoeotic mutation affecting antennae and legs. Drosoph. Inf. Serv..

[48] Siegrist SE, Haque NS, Chen C-H, Hay BA, Hariharan IK (2010). Inactivation of Both foxo and reaper Promotes Long-Term Adult Neurogenesis in Drosophila. Curr. Biol..

[49] Worley MI, Alexander LA, Hariharan IK (2018). CtBP impedes JNK- and Upd/STAT-driven cell fate misspecifications in regenerating Drosophila imaginal discs. eLife.

[50] Brand AH, Perrimon N (1993). Targeted gene expression as a means of altering cell fates and generating dominant phenotypes. Development.

[51] Gehring W (1966). Übertragung und Änderung der Determinations qualitäten in Antennenscheiben-Kulturen von Drosophila melanogaster. Development.

[52] Haynie JL, Bryant PJ (1986). Development of the eye-antenna imaginal disc and morphogenesis of the adult head in Drosophila melanogaster. J. Exp. Zool..

[53] Ing T, Tseng A, Sustar A, Schubiger G (2013). Sp1 modifies leg-to-wing transdetermination in Drosophila. Dev. Biol..

[54] Pfeiffer BD (2008). Tools for neuroanatomy and neurogenetics in Drosophila. Proc. Natl. Acad. Sci..

[55] Hazelrigg T, Levis R, Rubin GM (1984). Transformation of white locus DNA in Drosophila: Dosage compensation, zeste interaction, and position effects. Cell.

[56] Halfon MS (2002). New fluorescent protein reporters for use with the drosophila gal4 expression system and for vital detection of balancer chromosomes. genesis.

[57] Spradling AC (1999). The Berkeley Drosophila Genome Project gene disruption project: Single P-element insertions mutating 25% of vital Drosophila genes. Genetics.

[58] Gouge, C. A. & Christensen, T. W. Detection of S Phase in multiple Drosophila tissues utilizing the EdU labeling technique. *Dros. Inf. Serv*. **93**, 203–212 (2010).

