# Supplementary material for: Cell fate changes induced by a Distal-less enhancer-trap transgene in the Drosophila antennal imaginal disc
Source: Sci Rep. 2018 Mar 21;8:4950. doi: 10.1038/s41598-018-23093-z (PMC5862905; doi:10.1038/s41598-018-23093-z)
Supplement: Supplementary file 1 — Supplementary Information [file 41598_2018_23093_MOESM1_ESM.pdf]

Cell fate changes induced by a *Distal-less* enhancer-trap transgene in the *Drosophila* antennal imaginal disc

Syeda Nayab Fatima Abidi and Rachel K. Smith-Bolton

Supplemental Figure 1

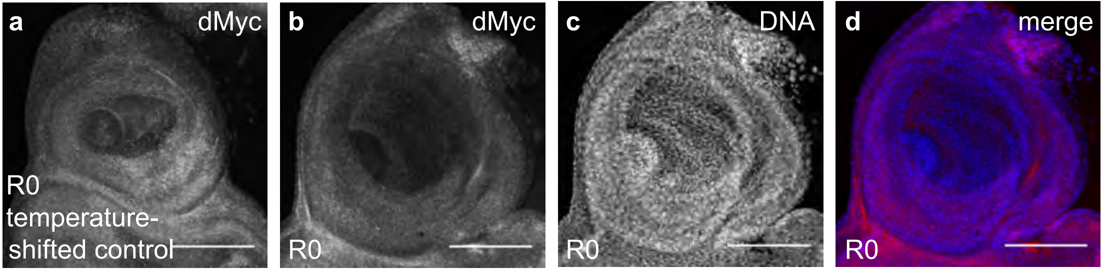

Supplemental Figure 2

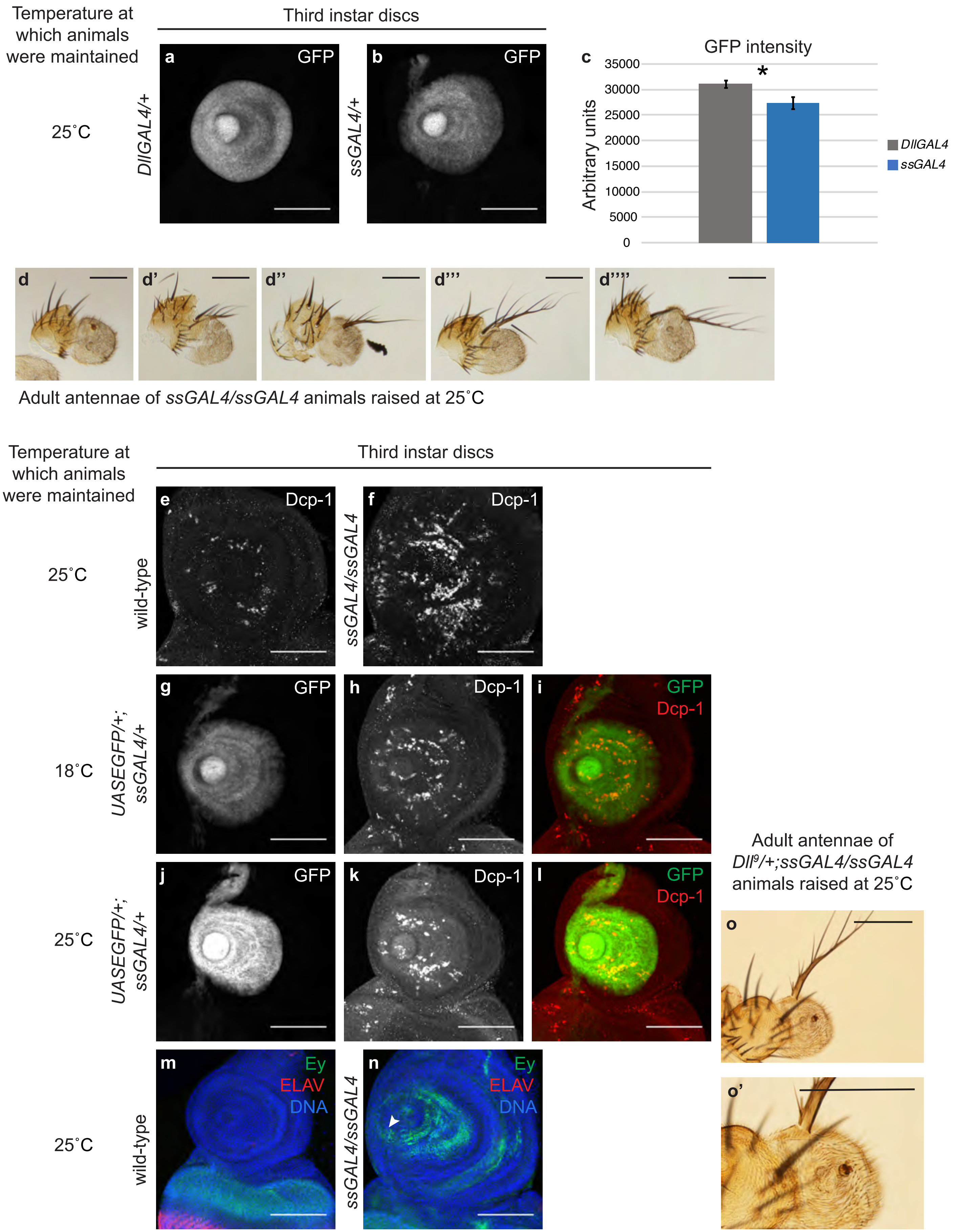

Supplemental Figure 3

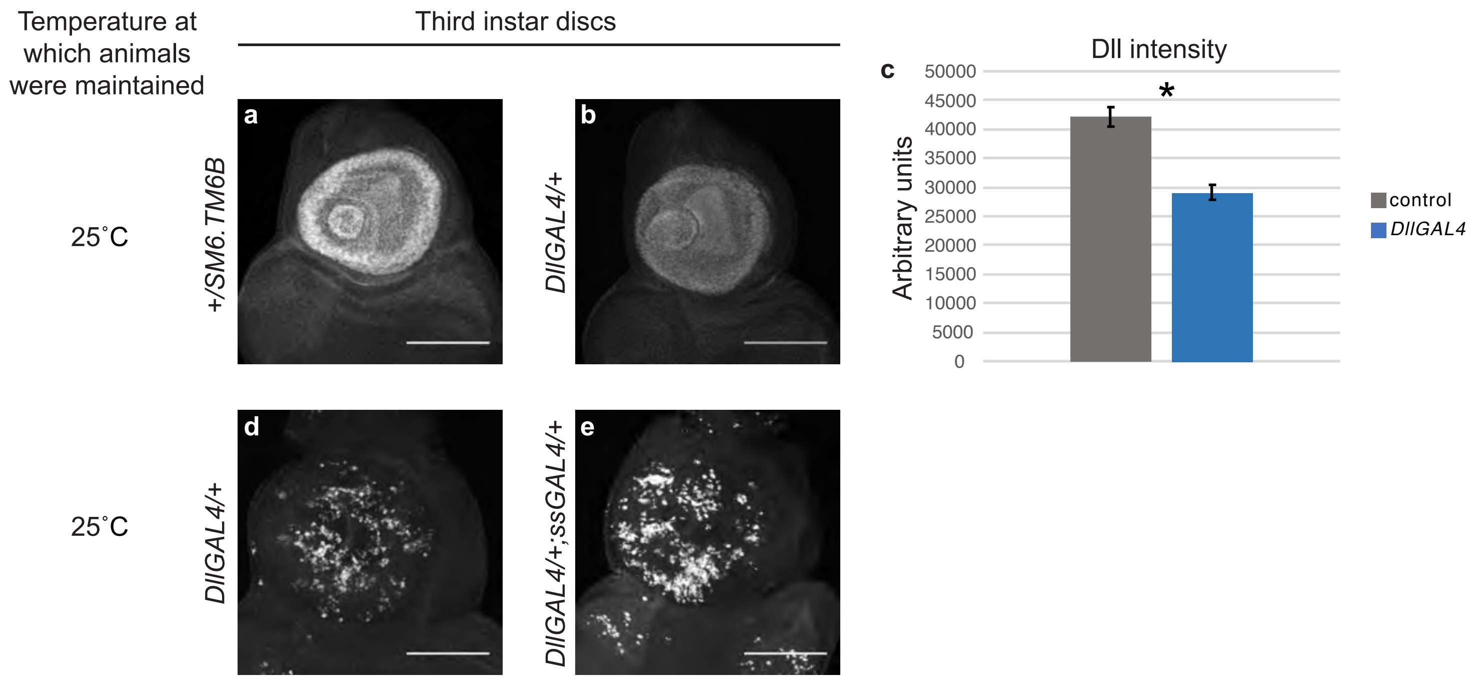

Supplemental Figure 1

Anti-Myc immunostaining in a temperature-shifted control disc (+;+/SM6.TM6B) at R0 (a) and an (*DllGAL4/*+) R0 disc (b). (c) DNA co-stain for the disc in (b) using TO-PRO-3. (d) Merge of (b) and (c) showing Anti-Myc (red) and DNA (blue). Scale bars are 100 µm.

Supplemental Figure 2

(a-b) GAL4 expression marked by UAS-EGFP in third instar discs at 25˚C. (a) *DllGAL4* expression. (b) *ssGAL4* expression. (c) Quantification of GFP intensity of *DllGAL4* (n = 15) and *ssGAL4* (n = 15). * p = 0.01. (d'-d'''') Adult antennae from ssGAL4 animals raised at 25˚C. The antennae showed a range of altered morphology. Similar to the DllGAL4/+ antennae, the arista and the third antennal segment were the most affected. (e-f) Anti-Dcp-1 immunostaining in a third instar control wild-type disc (e) and a homozygous ssGAL4 disc (f) maintained at 25˚C. Note that the *ssGAL4* disc shows much higher levels of apoptotic nuclei. (g-i) Third instar disc from a ssGAL4/+ animal maintained at 18˚C. (g) ssGAL4 expression marked by UAS-EGFP, (h) Dcp-1 immunostaining, (i) merge of (g) and (h). (j-l) Third instar discs from ssGAL4/+ animals maintained at 25˚C. (j) ssGAL4 expression marked by UASEGFP, (k) Dcp-1 immunostaining, (l) merge of (j) and (k). While GAL4 activity increased with the increase in temperature, as observed from the GFP intensity (g and j), cell death did not appear to be affected by the increase in temperature (h and k). (g-l). Anti-Ey immunostaining (green) and ELAV immunostaining (red) in third instar control wild-type discs (m) and homozygous ssGAL4 discs (n) maintained at 25˚C. Images were taken with the same confocal settings. Note that the Ey staining in (n) is not present near the weak point,

arrowhead in (n). (o-o') Adult antennae of Dll9/+; ssGAL4/ssGAL4 animals raised at 25˚C. (o') Higher magnification of (o). Student's T-test used for statistical analysis. Scale bars are 100 µm.

Supplemental Figure 3

(a-b) Anti-Dll immunostaining in third-instar control *+/SM6.TM6B* discs (a) and *DllGAL4/+* discs (b) maintained at 25˚C. (c) Graph showing quantification of Dll immunofluorescence intensity for *+/SM6.TM6B* discs (n = 10) and *DllGAL4/+* discs (n = 11). Note that *DllGAL4* discs show much lower levels of Dll. * p = 0.000005. (d-e) Anti-Dcp-1 immunostaining in third instar *DllGAL4/+* discs (d) and *DllGAL4/+;ssGAL4/+* discs (e) maintained at 25˚C. Student's T-test used for statistical analysis. Scale bars are 100 µm.

Supplemental Tables

Supplemental Table 1. Frequencies of antenna-to-eye fate change in different genotypes.

Genotype	Antenna-to-eye fate change	Temperature
DllGAL4/+	6-14% (range observed from 4 independent experiments)	30 ˚C for 24 hours
DllGAL4/+	9-27% (range observed from 7 independent experiments)	25 ˚C
DllGAL4/CyO	4 in 168 antennae (2.4%)	25 ˚C
DllGAL4/CyO, Gal80	0 in 488 antennae	25 ˚C
Dll^{5}/+	0 in 206 antennae	25 ˚C
Dll^{0109}/+	0 in 224 antennae	25 ˚C
Dll^{9}/+	0 in 258 antennae	25 ˚C
ssGAL4/ssGAL4	0 in 334 antennae	25 ˚C
Dll^{01092}/+; *ssGAL4/+*	0 in 290 antennae	25 ˚C
Dll^{9}/+; *ssGAL4/+*	0 in 302 antennae	25 ˚C
Dll^{5}/+; *ssGAL4/+*	0 in 470 antennae	25 ˚C
Dll^{9}/+; *ssGAL4/ssGAL4*	0 in 260 antennae	25 ˚C
Dll^{5}/+; *ssGAL4/ssGAL4*	0 in 232 antennae	25 ˚C
DllGAL4/+;ssGAL4/+	0 in 442 antennae	25 ˚C

Supplemental Table 2. Frequencies of antenna-to-leg fate change in different genotypes.

Genotype	Antenna-to-leg fate change	Temperature
Dll^{5}	22 in 412 antennae (5.3%)	25 ˚C
Dll^{5}/+; *ssGAL4/+*	91 in 470 antennae (19.4%)	25 ˚C

Supplemental Table 3. Frequencies of necrotic tissue in different genotypes.

Genotype	Necrotic tissue	Temperature
Dll^{9}/+; ssGAL4/ssGAL4	8 in 260 antennae (3.1%)	25 ˚C
Dll^{5}/+; ssGAL4/ssGAL4	3 in 232 antennae (1.3%)	25 ˚C
